# Facilitators and barriers to physical activity following pulmonary rehabilitation in COPD: a systematic review of qualitative studies

**DOI:** 10.1038/s41533-018-0085-7

**Published:** 2018-06-04

**Authors:** Hayley Robinson, Veronika Williams, Ffion Curtis, Christopher Bridle, Arwel W. Jones

**Affiliations:** 1 Lincoln Institute for Health, University of Lincoln, Lincoln, UK; 2 Nuffield Department of Primary Care Health Sciences, University of Oxford, Oxford, UK

## Abstract

Pulmonary rehabilitation has short-term benefits on dyspnea, exercise capacity and quality of life in COPD, but evidence suggests these do not always translate to increased daily physical activity on a patient level. This is attributed to a limited understanding of the determinants of physical activity maintenance following pulmonary rehabilitation. This systematic review of qualitative research was conducted to understand COPD patients’ perceived facilitators and barriers to physical activity following pulmonary rehabilitation. Electronic databases of published data, non-published data, and trial registers were searched to identify qualitative studies (interviews, focus groups) reporting the facilitators and barriers to physical activity following pulmonary rehabilitation for people with COPD. Thematic synthesis of qualitative data was adopted involving line-by-line coding of the findings of the included studies, development of descriptive themes, and generation of analytical themes. Fourteen studies including 167 COPD patients met the inclusion criteria. Seven sub-themes were identified as influential to physical activity following pulmonary rehabilitation. These included: intentions, self-efficacy, feedback of capabilities and improvements, relationship with health care professionals, peer interaction, opportunities following pulmonary rehabilitation and routine. These encapsulated the facilitators and barriers to physical activity following pulmonary rehabilitation and were identified as sub-themes within the three analytical themes, which were beliefs, social support, and the environment. The findings highlight the challenge of promoting physical activity following pulmonary rehabilitation in COPD and provide complementary evidence to aid evaluations of interventions already attempted in this area, but also adds insight into future development of interventions targeting physical activity maintenance in COPD.

## Introduction

Chronic obstructive pulmonary disease (COPD) is a common and preventable condition, characterised by persistent respiratory symptoms and airflow limitation that is caused by significant exposure to noxious particles or gases.^1^ COPD is treatable, with the aim to manage symptoms and minimise disease progression; however, there is no cure. People with COPD have significantly lower levels of daily physical activity (PA) compared with age-matched healthy individuals,^2–7^ an avoidance of PA often related to exertional dyspnea which leads to increasing inactivity, muscle weakness and reduced exercise capacity. Physical inactivity predicts poor prognosis across the course of the disease,^8,9^ including reduced quality of life and increased risk of hospitalisation^10–13^ and mortality.^14^ As such, the importance of PA in COPD management is recognised, and there has been wide interest in strategies to promote and support PA.^1^

Pulmonary rehabilitation (PR) is defined as “a comprehensive intervention based on a thorough patient assessment followed by patient-tailored therapies that include, but are not limited to, exercise training, education, and behaviour change, designed to improve the physical and psychological condition of people with chronic respiratory disease and to promote the long-term adherence to health-enhancing behaviour”.^15^ The key benefits of PR include clinically important improvements in dyspnoea, physical capacity, and quality of life.^16^ Exercise capacity has been regarded as key to modifying PA in COPD.^17^ However, increased exercise capacity following PR does not directly translate to an increase in daily PA^15,18^ and long-term behaviour change in COPD. It has been suggested that longer-lasting PR programmes are more effective than shorter programmes in increasing PA levels but the effect remains controversial.^19^ Others who have aimed to increase PA through exercise training have only identified modest, short-term increases in PA.^18,20^ Based on current available evidence it appears exercise training alone is not enough to maintain PA in COPD.^15,20,21^

Evidence from a recent systematic review and meta-analysis identified only individualised or pedometer-based counselling added to multidisciplinary PR produce changes that exceed the established minimal clinical important difference in daily steps for COPD.^20^ The minimal efficacy of interventions surrounding PA and behaviour change following PR has been attributed to the limited understanding concerning the determinants of PA on a patient level in COPD.^14^ It has been acknowledged that it is likely not to be a “one size fits all” regarding strategies to maintain short-term benefits of PR,^22^ and widespread individual differences of PA in response to PR have been proposed.^17,23–25^

PA is a complex behaviour,^14,15,17,18,20,21,24,25^ but thus far syntheses of the research surrounding PA following PR is predominantly based on randomised controlled trials using quantitative methods alone.^18,20,21^ These methods do not capture individuals’ insights on how and why interventions did, or did not promote PA. The use of qualitative methods enables researchers to gain a comprehensive understanding of the effectiveness of health interventions, providing complementary data to quantitative findings in randomised controlled trials. Systematic review of qualitative studies have provided evidence toward understanding the COPD patients’ subjective view of the impact of PR,^24^ and the barriers and enablers to participation in structured PA (i.e., exercise/PR programmes).^25^ Such evidence does not address the factors which influence the maintenance of PA and long-term behaviour change following PR, where the focus shifts to self-management of PA. Specific factors are proposed to be involved in maintenance of behaviour, different from those involved in behaviour initiation;^26^ however, these factors have not been explored in this specific population.

There is a need for a better insight into the patient subjective experience of PA following completion of PR to inform future practice and policies surrounding support of long-term adherence to health-enhancing behaviour in COPD. The aim of this systematic review was to therefore provide a comprehensive synthesis of the patient reported facilitators and barriers of PA following completion of PR, among individuals with COPD.

## Results

### Study selection

**Fig. 1:**
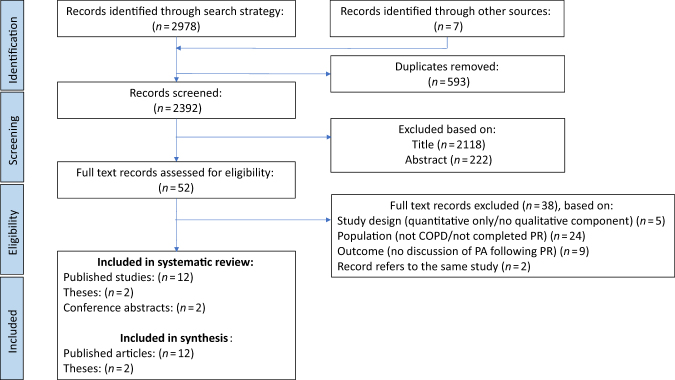
PRISMA flow chart representing the study screening process

Following removal of duplicates, searching identified 2392 records for eligibility assessment, of which 2340 were excluded based on title and abstract (Fig. 1). Full text screening of the remaining records resulted in 18 records that were eligible for the review. A full list of excluded studies, together with reasons for exclusion, can be found in the Supplementary File. However, only 14 studies were included within the synthesis (*n* = 12 published articles; *n* = 2 theses). In two cases, records referred to the same study^27–30^ and the remaining studies^31,32^ (*n* = 2) were presented only as conference abstracts. These were identified as relevant to the research question of this review, but not eligible for inclusion within the synthesis of the results due to lack of availability of participant quotations.^31,32^ Authors of these studies were contacted for more information; however, there was no response from authors.

### Study characteristics

Twelve studies were of a qualitative study design only, and two studies were of a mixed-method design^27,33^ (Table 1). Studies were conducted in Canada,^30,33,34^ Norway,^27,35,36^ The Netherlands,^37^ England,^38–41^ USA,^42,43^ and Sweden^44^ between 1998 and 2017. All studies collected data using either semi-structured interviews,^33–37,41,42,44^ open-ended interviews,^43^ or focus groups.^27,30,38–40^ The type of analysis ranged from thematic analysis,^27,30,34,35,39^ template analysis,^40^ qualitative content analysis;^37,44^ analysis methods informed by grounded theory,^38,41,42^ methods informed by phenomenology,^43^ and one reported that their analysis adhered to “established guidelines”.^33^

**Table 1:** Characteristics of included studies

Author (year), country	Design	Qualitative data collection methods; setting; analytical approach	Sample size (*n*)	Gender (M/F)	Age (years)	COPD characteristics	Pulmonary rehabilitation setting; duration; intensity	Data collection context: duration after pulmonary rehabilitation; participation in usual care or intervention; duration/intensity of the intervention
Camp et al. (2000), Canada^33^	Mixed methods	Semi-structured interview Participants home ^a^Established guidelines for data analysis	7	2/5	Range: 82–86	Severe to moderately severe (FEV1 % predicted mean (SD), 43 (14)), range = 23–69 Smoking (pack-years): mean (SD) 37 (21), range = 0–80	Hospital-based/in-patient PR 5 weeks 3 × weekly sessions	<2 weeks Usual care
Desveaux (2014), Canada^30^	Qualitative	Focus groups Setting: NR Thematic content analysis	12	6/6	Range: 52–85	Severe (FEV1 % predicted mean (SD): 44 (18)) MRC: median = 3	Hospital-based PR Inpatient PR: 6 weeks Outpatient PR: 12 weeks	>6 months Individuals were participating in community exercise maintenance programme 12 months/2 × 1 h sessions per week
Desveaux (2017), Canada^34^	Qualitative	Semi-structured interviews Participants rehabilitation hospital Deductive thematic analysis	6^b^	3/3	Range: 65–74	Number of comorbidities range: 1–6	Hospital-based PR	>3 months Usual care
Halding (2012), Norway^35^	Qualitative	Semi-structured interviews Participants’ homes or at the researcher’s office Thematic analysis	T1 = 18, T2 = 15^c^	13/5	Range: 52–81	Mild to severe Smoking status (*n*): Former = 11, Current = 5	Hospital-based PR Outpatient PR programme 12-week; (1 day/week)	T1 = <2 months, T2 = <12 months Usual care
Hoaas (2016), Norway^28,27^	Mixed methods	Focus group Rehabilitation centre Thematic analysis Systematic text condensation	10	5/5	Mean: 55.2 years	Moderate to severe Oxygen users (*n*): 3	Inpatient programme 4-weeks; (5 days/week)	T1: <18 months T2: <30 months T3: <42 months^d^ Tele-rehabilitation intervention 24 months/3 × 30 min per week
Hogg (2012), England^38^	Qualitative	Focus groups Community hospital Informed by grounded theory	16 Group A: 9 Group B: 7^e^	Group A: 4/5 Group B: 5/2	Mean (SD) Group A: 71 (10) Group B: 67 (11)	Mild to severe; FEV1 % predicted mean (SD): Group A: 67 (16); Group B: 59 (17) MRC: mean (SD): Group A: 2.1 (0.5); Group B: 2.3 (0.4)	Outpatient programme 8-weeks	<24 months Usual care
Lewis and Cramp (2010), England^39^	Qualitative	Focus groups Setting: NR Inductive thematic analysis	6	1/5	Mean: 69.3 years Range: 61–83	Moderate to very severe MRC: 2 (*n* = 5) 4 (*n* = 1)	NR	<48 months Usual care
Norweg (2008), USA^42^	Qualitative	Semi-structured interviews Interviews at home or rehabilitation centre Informed by grounded theory	4	1/3	Mean: 73 Range: 69–80	Disease length (years): 0.25–20 Oxygen users (*n*): 1	Outpatient programme 7.5 weeks^f^ 2× week	6–11 months Usual care
Rabinowitz (1998), USA^43^	Qualitative	Open-ended interviews Interviews at participants homes Informed by phenomenological approach	8	3/5	Mean: 64 Range: 45–75	Smoking status (*n*): former smoker (7), current smoker (1) Oxygen users (*n*): Supply at home as required (8) long-term (1)	In-patient programme 3 weeks; (3 × 1 h daily sessions)	<18 months Usual care
Rodgers (2007), England^40^	Qualitative	Focus groups Setting: NR Template analysis	23	14/9	Range: 63–70 years	FEV1 % predicted (range between focus groups 1–4): 40–49% MRC median: (range between focus groups 1–4): 3–4	Outpatient programme 6 weeks 2× week	<4 months Usual care
Stewart (2014), The Netherlands^37^	Qualitative	Semi-structured interviews Setting: assessment centre or patients’ home Qualitative content analysis	22	14/8	Mean (SD) 63.5 (7.8) Range: 45–78	Mild to very severe; FEV1 % predicted: Mean (SD): 52.5 (14.4) Disease length (years) mean (SD): 5 (3.9) MRC: mean (SD) 2.9 (1.3).	Outpatient programme 4 months	<8–11 months Participants were involved in an ongoing NUTRAIN trial (nutritional supplementation during 4 months of supervised exercise training)
Sundfør(2010), Norway^36^	Qualitative	Semi-structured interviews Participants homes Systematic text condensation	6	2/4	Mean: 64.5 Range: 55–75	Moderate to severe Disease length (years): 0.5–20	Hospital programme 4 weeks 1+ session daily	Between 4–6 months Usual care
Williams (2010), England^41^	Qualitative	Semi-structured interviews Participants homes Inductive approach informed by grounded theory methods	9	6/3	Range: 54–84	Moderate to very severe Disease length range (years): <5–>10 Oxygen users (*n*): 1	Outpatient 8 weeks 2× weekly	T0: interview pre-PR T1: interview post-PR; 1–2 weeks Usual care
Zakrisson (2014), Sweden^44^	Qualitative	Semi-structured interviews PHC and participants homes Qualitative content analysis	20	13/7	Mean (SD): 68 (4.1) Range: 62–78	Moderate to severe FEV1 % predicted Mean (SD): 46 (10) Smoking status (*n*): Current smoker (4)	PHC 6 weeks 2 h per week	<36 months^g^ Usual care

*n* number, *COPD* chronic obstructive pulmonary disease, *PR* pulmonary rehabilitation, *M/F* male/female, *FEV1 (% predicted)* percentage of forced expiration volume in one second divided by the average FEV1% in the population for any person of similar age, sex, and body composition, *SD* standard deviation, *NR* not reported, *PHC* primary health care, *GOLD stages* global initiative for chronic obstructive lung disease stages, *MRC dyspnoea* medical research council dyspnoea scale, *T1/T2/T3* time 1/time 2/time 3

^a^Analytical approach was: “established guidelines for data analysis”

^b^This study involved participants with heart failure but only the COPD subgroup was reported in this table

^c^Two people did not provide follow-up interviews because of death, and one could not be reached

^d^T3 refers to a second paper which followed up the same participants’ experiences of PA following tele-rehabilitation

^e^Using records held by the pulmonary rehabilitation team, eligible participants were placed into two groups (Group A: had received input from pulmonary rehabilitation staff to assist with ongoing exercise following completion of the pulmonary rehabilitation course; Group B: had not received any input from pulmonary rehabilitation staff regarding ongoing exercise)

^f^Estimated duration of PR programme, based on “six, 1-h weekly sessions of occupational therapy” and “15 sessions held twice weekly” of the exercise training programme

^g^Data collection timescale post-PR estimated from reported information: PR during: 2007–2008, interviews in Spring 2009

Overall, there were a total of 167 individuals diagnosed COPD across the studies (male = 92, female = 75). All participants had previously completed PR but the treatment varied on setting and duration. The duration of the PR ranged from 4–12 weeks and individuals attended either inpatient^27,30,33,34,43^ and outpatient PR venues^35,37,38,40–42^ or setting was not reported.^36,39,44^ Most individuals were not involved in PA maintenance interventions;^33–37,39–44^ however, some were involved in post-rehabilitation programmes, such as a 6-month community exercise maintenance programme,^30^ a 2-year tele-rehabilitation intervention,^27^ and approximately half of individuals within Hogg’s et al.^38^ study had received input regarding ongoing exercise programmes post-rehabilitation.^38^ The context of data collection after completion of PR also varied across studies. The data collection typically occurred at either the individuals’ home,^33,36,41,43^ the rehabilitation centre,^27,34,38^ or a combination of both^35,37,42,44^ and data collection settings were not reported in some studies.^30,39,40^ All but two of the studies collected data just once following PR. One study conducted two semi-structured interviews^35^ and the other conducted three focus groups.^27^ Data collection took place between 1–2 weeks and 42 months following completion of PR.

### Critical appraisal

Critical appraisal of the studies was conducted by two reviewers (Table 2). All studies were interpreted as having a clearly focused research question or hypothesis, as having an appropriate research design, appropriate justification of sampling strategy, well-described method of data collection, an explicit discussion of ethical issues and a consistent approach to reporting the conclusions (within the abstract and discussion). For each criterion, most studies were interpreted as good quality, with the exception of conflicts of interest and sponsorships, which were reported in six studies.^27,30,33,37,40,44^ In four studies it was unclear how well the researchers knew the participants,^30,34,35,42^ and in two studies no description of the relationship between participant and researcher was reported.^39,44^ In one study, the analytical method was not found to be clearly justified.^37^ The credibility of one study was unclear, due to minimal quotations to support themes.^35^ In two studies there was a lack of primary data provided within the text to support the deducted themes and subthemes from the study.^33,39^ In one study, the author did not identify limitations.^43^

**Table 2:** Critical appraisal of the included studies

	Camp (2000)^33^	Desveaux (2014)^30^	Desveaux (2017)^34^	Halding(2012)^35^	Hoaas (2016)^27,28^	Hogg (2012)^38^	Lewis and Cramp (2010)^39^	Norweg (2008)^42^	Rabinowitz (1998)^43^	Rodgers (2007)^40^	Stewart (2014)^37^	Sundfør (2010)^36^	Williams (2010)^41^	Zakrisson (2014)^44^
Clearly focused question/hypothesis?	+	+	+	+	+	+	+	+	+	+	+	+	+	+
Is the choice of qualitative method appropriate?	+	+	+	+	+	+	+	+	+	+	+	+	+	+
Is the sampling strategy clearly described and justified?	+	+	+	+	+	+	+	+	+	+	+	+	+	+
Is the method of data collection well described?	+	+	+	+	+	+	+	+	+	+	+	+	+	+
Is the relationship between the researcher(s) and participants explored?	+	?	?	?	+	+	−	?	+	+	+	+	+	−
Are ethical issues explicitly discussed?	+	+	+	+	+	+	+	+	+	+	+	+	+	+
Is the data analysis/interpretation process described and justified?	+	+	+	+	+	+	+	+	+	+	?	+	+	+
Are the findings credible?	−	+	+	?	+	+	−	+	+	+	+	+	+	+
Are there any sponsorships/conflicts of interest reported?	+	+	−	−	+	−	−	−	−	+	+	−	−	+
Did the authors identify any limitations?	+	+	+	+	+	+	+	+	−	+	+	+	+	+
Are the conclusions the same in the abstract and discussion?	+	+	+	+	+	+	+	+	+	+	+	+	+	+

(+) = yes; (-) = no; (?) = unclear

### Data synthesis

**Fig. 2:**
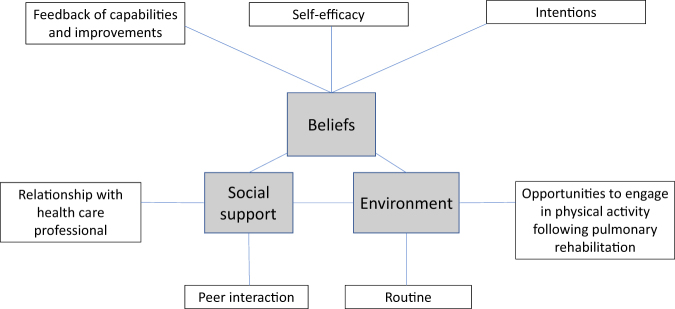
Concept map to illustrate the analytical themes and sub-themes relating to physical activity maintenance following pulmonary rehabilitation in COPD

During the synthesis of the data from the 14 studies included in this review, 7 sub-themes were identified as influential to PA following PR, which were organised into 3 analytical themes, including beliefs, social support, and environment. The theme “beliefs” has three sub-themes, including intentions, self-efficacy, and feedback of capabilities and improvements. The theme “social support” has two sub-themes, including relationship to health care professionals and peer interaction and the final theme “environment” also has two sub-themes, including opportunities following PR and routine (Fig. 2). Facilitators and barriers to PA within these analytical themes are presented within Table 3, alongside a selection of participant quotes from included studies to reflect these themes. A line of argument, depicting the key analytical themes and sub-themes are reported.

**Table 3:** Analytical themes and sub-themes with reference to quotations within primary studies

Beliefs	Social support	Environment
**Intentions** Information and education within PR influences attitude toward PA^35–38,40,42,44^ (+/−) “I became aware that I need not feel so frightened when out of breath and that was the most important. I felt that I got sufficient information to make me calm and less frightened when I lose my breath”^44^ Beliefs surrounding PA and health influences intentions -PA is enjoyable/associated with health benefits^27,35,37,42,43^ (+) “I have always enjoyed [exercising]. Only the cycling I don’t really enjoy, especially in the winter. But I realise now that particularly cycling means a lot to my physical condition and it would’ve been worse if I hadn’t cycled”^37^ -PA is difficult/uncertainty regarding health benefits:^36,38,40^ (−) “I went and bought a bike, it’s downstairs. Bike is hard, bike is hard if you don’t know how to do it and how much to do it”^38^ **Self-efficacy** -Previous experiences influence beliefs of capability and confidence: (+/−) -Exacerbations and symptoms lead to psychological distress^27,30,35,36,38–40,43^ (−) “Come out of hospital and just feel and just feel sorry for yourself and not want to do anything”^39^ -Breathlessness is associated with anxiety^35,36,38,43^ (−) “… I don’t want to go through things like that, if I can avoid it. I get nervous when I feel I can’t breathe, it’s scary, it gets very scary”^43^ -Confidence to apply breathing and self-management techniques^33,35,36,38,41,42^ (+) “I saw the light one day. I was using the oximeter while cleaning the house, and I discovered that I was sooo low. I didn’t use the oxygen while doing housework before, but I do now”^27^ **Feedback of capabilities and improvements** Awareness of change/self-monitoring health influences motivation (+/−) -Recognition of improvements increased motivation to be active:^27,30,37,38,41–44^ “… I’m doing my own hoovering which I wasn’t doing because he did it, I’m cleaning windows, which he did, I do, you know there is, yeah, definitely sharing the jobs more” “ … definitely doing more than I was ……”^41^ -Noticing health decline/lack of upward feeling negatively affected individuals outlook on PA:^27,43^ (−) “When you don’t see results, you kinda say, ahhh… I don’t know”^43^ -Self-monitoring allowed individuals to acknowledge capabilities^27,42^ (+) “I have become more aware of what I do through registrations of my workouts. I am more engaged in my own health”^27^	**Relationship with healthcare professionals** -Contact regarding PA maintenance groups led to participation in weekly activities^35^ (+) “The physiotherapist called me, and she asked me if I would like to continue with a group. Now I exercise with them every Thursday. I have planned to join other activities as well!”^35^ -Delivery of information via leaflets and the internet about PA was difficult to follow/not well accepted^40^ (−) *“*But I do find leaflets what you’ve been given here or there they’re not exactly in plain English and they do take a lot of understanding”^40^ -Provide a sense of security and comfort: helps overcome anxiety regarding symptoms^27,30,39^ (+) *“*It meant, I like the fact that they allowed you to learn or go at your own pace. Nobody’s pushing or pulling”^30^ *“*One of the best things with the project has been to meet the [tele-]physiotherapist once a week, and get to ask questions about everything that is on your mind^27^ -Continued support after PR regarded as beneficial^27,30,34,35,37,38,42–44^ (+) “They give you confidence … to push yourself a bit, to try to do a bit more”^38^ -Support ends after PR negatively affects PA participation:^34,38,43^ (−) “I don’t have the incentive and I don’t have anybody to kick my ass and tell me to get it done”^34^ **Peer interaction** -Provide a sense of solidarity and support after PR:^27,30,34,35,37–40,43,44^ (+) “The people that I know at the gym, we’ve all done pulmonary rehab and we all have a cup of tea after we exercise together and that encourages me to go, cos I think ‘Ooh if I don’t go today … they’ll wonder where I am"^38^ Peer interaction within PR affected individuals’ confidence following PR: Pre-PR: “[I do] nothing really, only stopping in the house really and listen to the radio and television”^41^ Post-PR: “… they have given me more confidence by being with people and going out twice a week for about 3/4 h, go there and come back you know, and meet people”^41^ -Individuals struggle when peer interaction ends:^35,38,43^ (−) “Exercises are all right in groups. However, to do it on your own …. I guess I don’t manage”^35^ -Reminder of disease progression is uncomfortable/can lead to avoidance:^35,39,43^ (−) “The meetings wouldn’t do me any good right now. I would feel like that could be me, you know, getting that bad—I don’t want to give in, so I feel it would drag me down more”^43^	**Opportunities to engage in PA following PR** -Individuals feel the need for clear information regarding exercise groups post PR^34^ (+) “Just to know where these places are would be a big benefit and how to get into them”^34^ -Individuals want and appreciate access to structured maintenance sessions after PR^34,38,39^ (+) “The best thing for me would be a mini-program, like we had in rehabilitation”^37^ -Access to maintenance sessions affected individuals’ participation in PA:^30,34,38,43^ (−) -Individuals were not motivated to exercise at home^38^ (−) “… it’s just difficult to get the motivation to do it at home”^38^ -Cost and proximity to sessions^30^ (−) “… I have to go a little bit of a distance to get there, which I’m quite willing to do, if it isn’t going to cost me money. But I want something closer to home if I have to do this on a regular basis, which I do”^30^ -PA venue is important; hospital-based programmes regarded as safe/supportive as they are associated with the health care system^34^ (+) *“*Because you know that the healthcare system is interested in what’s going on with your exercise program^34^ -Social environment of the PA venue is important. Public gyms can feel intimidating^27^ (−) “If I want to go to the gym, it is a 60 km drive from my house. Moreover, I would have felt weak in front of others. They would have looked at me, and thought: He cannot do anything …”^27^ -Social isolation can be a barrier to managing negative emotions^30,35,39,40^ (−) “Exercises are all right in groups. However, to do it on your own…. I guess I don’t manage”^35^ -Access to preferred activity influences intentions and motivation to be active^37^ (+) “The cycling not so much, I do that because I have to, but the walking. I enjoy walking a lot. I don’t need motivation to do that”^37^ **Routine** -Establishing routine after PR facilitates PA maintenance:^27,36,38,42,44^ (+) “It’s like I get up, I brush my teeth, I get dressed and I get on the treadmill before I even go downstairs … I know if I’m going to do it, I’ve got to get into a routine …”^42^ -Family understanding of importance of PA:^37^ (+) “If my husband wouldn’t have been here, I would’ve needed help at home because I couldn’t manage alone. He also stimulates me to exercise”^37^ -Home responsibilities; caring for partner limits PA opportunities:^44^ (−) “Now my husband is at home all the time, and since last winter when he fell ill he is too weak to go for walks so this winter there haven’t been any”^44^ -Negative pressure from relatives and family leads to avoidance of PA^27,43^ (−) *“*They’re always yelling at me, Ma, you know. And I say, Leave me alone, I don’t tell you what to do, don’t you tell me what to do”^43^ -Combination of both means individuals fall back into old habits:^35,36,39,43^ (−) “Anyway, you will fall back to the old way of doing it. Because you have done so many times. It is difficult”^36^

(+) = facilitators; (-) = barriers

### Theme: beliefs

Thoughts surrounding the importance of PA, previous experiences prior to PR, recognition of improvements following PR, and confidence influence individuals’ intentions and motivation toward maintenance of PA following PR.

### Sub-theme: intentions

Many individuals held the belief that PA was enjoyable and important for their physical and psychological well-being.^27,35,37,42,43^ Individuals’ beliefs toward PA often manifested into intentions to be more physically active.^27,35,37,42,43^ These beliefs were often influenced by individuals’ lifestyles prior to COPD diagnosis. For example, regular engagement in PA prior to COPD diagnosis facilitated positive intentions toward maintaining PA following PR.^37,43^ Information and education on PA was also identified as a facilitator to PA following PR, as it increased individuals’ understanding of the health benefits associated with PA.^35–38,40,42,44^

Intentions to be more physically active did not always translate into behaviour change. For example, in one study, individuals repeatedly indicated positive intentions toward PA following PR;^43^ however, they were labelled as non-exercise compliant, highlighting an intention-behaviour gap in PA.

### Sub-theme: self-efficacy

Self-efficacy was a common theme throughout the data, referring to individuals’ beliefs in their ability to engage in PA. A barrier to PA maintenance was negative beliefs surrounding PA and health.^36,38,40^ These negative beliefs often stemmed from individuals’ previous experiences of PA that negatively affected their confidence and perceived capability of engaging in PA following PR. For example, individuals reported PA as too hard and that they were too restricted by symptoms such as breathlessness.^38^ Exacerbations and symptoms often led to psychological distress, for example, individuals reported feeling overwhelmed, saddened, and frustrated by their restrictions due to COPD,^27,30,35,36,38–40,43^ and these experiences negatively influenced individuals’ confidence to be active. Breathlessness and anxiety were repeatedly reported throughout the studies,^35,36,38,43^ and this anxiety represented a barrier to PA after PR when individuals were attempting to maintain PA at home or by themselves, especially when individuals felt socially isolated.^35,43^ Avoidance was a strategy developed to manage anxiety associated with breathlessness^27,38,43^ and individuals did not want to draw attention to themselves by exercising outside of the house.^27^ However, confidence to apply stress and breathing management techniques was often reported a facilitator to maintaining PA after PR.^33,35,36,38,41,42^ These skills were often learnt during PR, and were associated with feelings of increased self-efficacy and a sense of empowerment,^27,41^ and the newfound confidence also coincided with a more positive outlook on life.^33^

### Sub-theme: feedback of capabilities and improvements

Feedback refers to monitoring and providing informative or evaluative information on the performance of PA behaviour.^45^ When individuals noticed their personal improvements, or recognised their capabilities,^42^ they were often more engaged or motivated by the outcomes, and felt empowered to maintain PA.^27,30,37,38,41–44^

This motivation was facilitated by long-term feedback from health professionals, for example, in a maintenance tele-rehabilitation study^27^ individuals reported that they had improved throughout the course, and felt a sense of accomplishment when discussing their progress, reporting that an upward feeling was important in motivating them to be active.^27^ Those who had noticed improvements in health wanted these benefits to be maintained, and reported that they wanted the exercise classes to continue.^38^ Positive feedback therefore promoted beliefs of improvement and encouraged individuals to stay active following PR. However, not recognising improvements was perceived as a barrier to PA maintenance, as individuals became unmotivated as they believed that the exercises were not worthwhile or helpful.^27,43^

### Theme: social support

Other people played a significant role in individuals’ journeys following PR. The perception of feeling cared for, valued, and assisted within the home and the community were important, in addition to relating to others who are in similar situations.

### Sub-theme: relationship with health care professionals

Individuals’ relationships with others had a large impact on their outlook and PA behaviour following PR, which extended beyond gaining feedback about their condition. Support from health care professionals was commonly reported as being important for individuals, whereby their authority instilled a sense of trust, and individuals felt safe and comforted by their presence after PR.^27,30,34,35,37,38,42–44^ Individuals were less fearful of being overwhelmed by their symptoms, were comforted by the opportunity to ask questions, and were encouraged by their interest in their personal health.^34^ A barrier to PA was the lack of maintained support from health care professionals, and individuals reported feeling unmotivated,^27,34,38,43^ for example, by a lack of encouragement, incentive, and uncertainty regarding transferring these exercises to a different environment, for example, to their home.^38^

### Sub-theme: peer interaction

Interaction with peers was commonly reported as beneficial and it was considered a facilitator to the maintenance of PA, as it made PA more enjoyable and helped individuals conquer feelings of loneliness.^27,30,34,35,37–40,43,44^ The opportunity to discuss symptoms and compare notes with others in similar situations also helped reduce distress associated with symptoms. However, individuals reported having a sense of loneliness that was difficult to manage following PR,^30,39,40^ and a lack of peer interaction following PR was considered a barrier to PA.^35,38,43^

Individuals appreciated the ease of connecting with peers, even when in different counties.^27^ Throughout the studies, interest was expressed in the maintenance of interaction with peers with COPD after PR,^35,38,43^ but was also expressed in socialising with individuals with mixed conditions and other members of the community.^30^ Despite this, peer interaction was also recognised as a barrier to PA, as it elicited fear in individuals as others’ conditions were an unwanted reminder of the progressive nature of COPD.^35,39,43^ This response motivated individuals to avoid peers in attempt to deny the illness and return to normality.^39^

### Theme: environment

Individuals’ surroundings influenced their opportunities to engage in PA following PR. In addition, individuals’ physical and social environment influenced their experiences and approach to maintaining a PA routine and successfully establishing habits following PR.

### Sub-theme: opportunities to engage in PA following PR

Individuals often expressed the importance of structured and unstructured PA sessions after PR,^34,38,39^ in particular they would like access to PA maintenance.^34,35^ However, unclear information regarding maintenance sessions did not allow for individuals to consider alternative ways to be active, and was considered a barrier to PA.^40^ Barriers to attendance in maintenance sessions also involved issues surrounding cost and proximity^30^ and restrictions imposed by family and work responsibilities.^44^ There was mixed views on home exercises, with some individuals reported positively to them,^27^ whereas others did not feel like they would be helpful,^38^ reflecting individual differences in preferences of PA. Walking and cycling^37^ were considered enjoyable activities, as well as simply being outside to enjoy the scenery.^43^ Individual differences regarding their preferred PA meant that having various opportunities to engage in a variety of activities was therefore considered a facilitator to PA, whereas restricted choice was considered a barrier to PA.

### Sub-theme: routine

Ongoing contact with health care professionals and peers through maintenance groups also provided a sense of structure following PR and the expectation and pressure to conform to pre-set times and activities was appreciated and regarded as a facilitator to maintaining PA.^27^ However, without access to ongoing structured PA sessions, individuals largely reported that a barrier to maintaining PA was falling back into old habits.^35,36,39,43^ Routine was considered an important facilitator by individuals throughout the studies.^35,38,42,44^ However, this routine was influenced by the individuals’ home life. For example, families’ understanding of COPD and their recognition of the importance of PA was identified as a facilitator to PA as they were able to provide support and encouragement,^37^ whereas attention sometimes added too much pressure to individuals dealing with COPD.^27,43^ For example, individuals did not appreciate their family telling them to exercise.^43^ Social isolation due to restricted access to structured PA groups, lack of motivation,^38^ as well as simply forgetting to be active^43^ were also all reasons why individuals fell back into their previous routines. In addition, establishing a successful routine was considered a long process which required patience,^36^ placing emphasis on the challenge faced by many individuals to avoid falling back into old habits established prior to PR.^27,35,40,42^

## Discussion

### Summary of the findings

The purpose of this review was to identify the patient reported facilitators and barriers of PA following PR. The analytical themes developed were beliefs, social support, and environment that encapsulated the identified patient reported facilitators and barriers of PA following PA. Key facilitators identified within this review were the perception of continued support from health care professionals, continued peer interaction, the sense of accomplishment gained through self-monitoring and feedback, as well as opportunities to access PA maintenance groups following PR that enabled individuals to form routines and establish habits. Key barriers to PA were symptoms that evoked anxiety and fear, for example, breathlessness upon exertion, restricted access to social support and structured maintenance sessions following PR, and lack of positive feedback regarding health which led to individuals being less likely to establish routines incorporating PA, and were more likely to return to previous habits formed prior to PR.

### Strengths and limitations of this review

Extensive searches for existing and ongoing systematic reviews suggest that there are no other systematic reviews to synthesise qualitative studies of COPD patients’ experiences of PA following PR. This systematic review followed a pre-specified protocol, conducting a comprehensive search strategy that yielded 14 studies. Language, date, and publication restrictions were not imposed in the search strategy, unlike previous qualitative systematic reviews in relevant areas, whereby inclusion criteria was either restricted to articles published in English^25,46^ or a selection of languages^24^ and included only peer-reviewed articles.^24,25,46^ Unpublished data proved valuable in this review, with a large amount of original qualitative data that provided clear insight into patients’ perspectives regarding PA following PR. We therefore consider inclusion of unpublished evidence to be a key strength of this review and suggest systematic reviews of qualitative studies that do not adopt this approach are at risk of publication bias and excluding data relevant to their research question. The resulting context within the included studies was diverse in terms of individuals’ PR settings, PA experiences following PR, and the cultural setting within each country, meaning that it was possible to achieve a higher level of abstraction in the synthesis.^47^ Two records retrieved in our search strategy were conference abstracts,^31,32^ and based on the available information were deemed to meet the inclusion criteria. It may be considered a limitation that the data from these studies are not included in our synthesis; however, as findings largely reflected existing themes we feel access to the full studies is unlikely to change the conclusions drawn in this review.

Our approach to data (thematic) synthesis were in line with established methodology for systematic reviews of qualitative evidence,^48^ an interpretative approach which enables a summary of the descriptive themes from the primary studies, with a subsequent production of analytical themes through applying a higher-level theoretical framework to answer the research question. This approach was beneficial, as transparency between the developed themes within this review and the text from the primary studies was maintained. It has previously been suggested that qualitative synthesis such as meta-ethnography can de-contextualise the findings from the primary studies.^49,50^ However, efforts to preserve context, in line with previous methods in thematic synthesis,^48^ were taken to consistently refer to primary studies to check for contextual factors that could affect transferability. Additionally, by adoption of key principles of systematic reviews (extracting and tabulating study characteristics), facilitators and barriers reported in included studies can be considered alongside their specific clinical and methodological characteristics.

Like quantitative research, there are no standardised criteria for assessing the quality of all qualitative research.^51^ This systematic review yielded studies with varied designs, methodological and analytical approaches, meaning that a key challenge was assessing the quality of research. We adopted an approach of appraising study quality by assessment of study conduct using a previously used critical appraisal tool.^52^ In accordance with approaches in systematic reviews of quantitative evidence, and with limited evidence to suggest that quality of reporting is associated with the credibility and transferability of the findings in qualitative studies,^53^ we did not feel there was sufficient justification for exclusion or weighting of study data according to quality. For each criterion within our chosen critical appraisal tool,^52^ the majority of the studies were critically appraised favourably; however, the limitations in some of the included studies should be considered, for example, credibility was often jeopardised by the small amount of qualitative data provided throughout the studies and it was not possible to conclude whether selected quotes were biased toward researchers pre-existing views regarding their research question.

### Comparison to previous reviews

No previous systematic review has synthesised qualitative data regarding facilitators and barriers to PA following PR. Meta-analyses of randomised controlled trials have provided limited success in demonstrating efficacy of interventions to improve PA in COPD, as effects are typically modest and short term.^18,20^ However, current proposals of the likely greater impact of longer duration PR programmes on modification of PA^19^ would support a key theme presented in our review, namely, that ongoing support from both health care professionals and peer interaction was a facilitator to PA maintenance. The importance of social support has previously been reported in systematic reviews researching individuals’ participation in PR^24,25^ and facilitators and barriers to PA in other lung conditions.^46^ Feeling supported by family throughout PR has been identified as a facilitator to PA during PR,^24^ but the results from this study suggest that family, friends, partners, and peers interaction are also important in the maintenance of PA.

During PR, it has previously been suggested that environmental and personal factors, in addition to social factors, have been recognised as influential to PA in patients with COPD.^25^ Environmental factors such as transportation and options regarding the type and intensity of PA were also considered influential factors to PA maintenance, as were personal factors associated with identity, for example, previous experiences with PA and previous lifestyles which affected individuals’ PA beliefs. In this review, a larger emphasis was placed on access to information regarding opportunities to engage in PA following PR, likely due to the responsibility of PA maintenance being shifted from health care professionals to patients after completing PR. Establishing a healthier routine is often reported to be a key benefit during PR;^24^ however, the findings from this review identified both the importance and difficulty of maintaining these PA routines and forming habits following PR. Interestingly, the length of the PR programme has previously been recognised as a barrier to PA,^25^ with less patients being likely to attend longer programmes; however, the findings in this review suggest that a barrier to PA maintenance is the loss of structure and contact with other people.

Unlike previous findings of key barriers to PR,^25^ smoking status was not identified as a barrier to PA maintenance in this systematic review. It may be argued that a greater proportion of COPD patients included in the present review (i.e., who completed PR) were less likely to be smokers who are associated with poorer completion rates in PR and hence have other important personal factors. Our findings suggest that COPD patients often reflect on their health. Positive feedback regarding PA and health was recognised as a facilitator to PA maintenance, whereas some became unmotivated if they did not recognise any improvements in their condition. This suggests that self-efficacy is an influential factor in PA motivation, complementing findings of previous systematic reviews that reported goal achievement as a facilitator to PA during PR, and the benefits of adding pedometer-based counselling to multidisciplinary PR.^20,25^

### Implications for research and practice

This systematic review furthers our understanding regarding the key factors that influence PA maintenance following PR in COPD and it provides evidence for health care professionals to consider when discussing individualised self-management plans on discharge from PR. Likewise, given that a typical pathway following PR includes referral of patients back to their primary care providers, this review provides important information for clinicians and healthcare professionals in these settings to consider when delivering long-term COPD management. Our findings provide complementary evidence to aid evaluations of PA interventions already attempted in this area,^18–21^ but also adds further insight into future development of interventions targeting PA maintenance in COPD. Based on the available evidence, further trials in this area should consider intervention functions which promote maintenance of social support from the health care professionals, maintenance of peer interaction following PR, promote a range of opportunities after PR to cater various PA preferences, and optimise individuals’ opportunities to recognise improvements in their health through techniques such as feedback and counselling to boost self-efficacy. This can be achieved by exploiting the use of wearable devices such as pedometers or through the use of information and communication technologies (tablet computers, smartphone applications, websites).

This systematic review further highlights the large variance of individual differences within the population who complete PR, supporting the statement that there is not a “one size fits all” approach to achieving lasting behaviour change following PR.^22^ This should be considered when tailoring interventions to individual patient needs.^21,23,54^ Alternatively, interventions with multiple components should be considered in targeting behaviour change, as they have the potential to influence PA maintenance by targeting various influential factors. Our findings have general applicability to all COPD patients completing PR, but researchers should also be mindful of context-specific factors, which could influence PA behaviour following PR in local COPD populations.

It is recommended that future research should adopt mixed methods designs, incorporating qualitative methods within randomised controlled trials to provide a more comprehensive evaluation of the factors influencing the efficacy of their interventions but also provides evidence as to how such interventions can be implemented in practice. Future qualitative research should consider and build upon the identified limitations within the studies included in this review. For example, transparency in the relationship between researcher and individual, description of study limitations, and access to participant quotations would further increase the credibility of the research in the area. It would be prudent for future studies in the area to therefore adhere to the Consolidated Criteria for Reporting Qualitative Research that facilitate critical appraisal and interpretation.

## Conclusions

This systematic review identified and synthesised the data referring to the patient reported facilitators and barriers to PA following PR, which provided an in-depth understanding and insight into patient experiences regarding maintenance of PA behaviour. The results from this systematic review highlight the complexity of behaviour change, and the challenge of promoting PA following PR on a population level. The results provide clear guidance for future research design, as well as recommendations regarding the content of future interventions.

## Methods

### Protocol

The protocol for this review was registered on the international prospective register of systematic reviews (PROSPERO) (CRD42017058274). This review was reported in accordance with the Preferred Reporting Items for Systematic Reviews and Meta-Analyses (PRISMA)^55^ guidelines and Enhanced Transparency of Reporting the Synthesis of Qualitative Research framework.^51^

### Eligibility criteria

Study design: Qualitative studies (interviews, focus groups) or mixed-methods designs which included qualitative data.

Participants: Adults with a diagnosis of COPD who have completed PR.

Exposure: Discussion of PA, which was defined as any bodily movement produced by skeletal muscles that results in energy expenditure,^56^ for example, experience of structured exercise such as fitness classes or walking groups, to activities of daily living such as shopping, meal preparing, or housework following PR.

Outcomes: Facilitators and barriers to PA following PR in COPD.

### Searching

A comprehensive search strategy was used between February 2017 and October 2017 to identify all relevant available studies. DARE, PROSPERO, Cochrane Airways, and Cochrane Database of Systematic Reviews were searched for ongoing and published reviews. For published original studies the following databases were searched: MEDLINE, Embase, Web of Science, CINAHL, ASSIA, PsycINFO, and SPORTDiscus. An example of a full search strategy for one database (MEDLINE) is provided in the Supplementary File. Database searches were also supplemented with internet searches (e.g., Google Scholar) and contact with study authors and experts when required. Forward and backward citation tracking from included studies and review articles were also conducted to identify relevant papers. ClinicalTrials.gov and Current Controlled Trials were searched for completed and ongoing trials. DART Europe E theses, EThOS, Open Grey, The New York Academy of Medicine, ProQuest Dissertations, theses.org, and Conference Proceedings Citation Index (Web of Science) were searched for unpublished data. All references were exported and stored in EndNote.

### Study screening

Two reviewers independently screened the titles and abstracts for inclusion against the defined eligibility criteria. Full-text articles were retrieved for articles that were not excluded based on title or abstract. Further independent screening of full texts was performed to determine eligibility with any disagreement between two reviewers resolved by consensus.

### Data extraction

Data from the included papers was extracted by two reviewers and was completed using a bespoke data collection form for qualitative research based on the UK National Institute for Health and Clinical Excellence (NICE) universal template.^57^ To facilitate synthesis of qualitative data, all studies were uploaded on to NVivo 11 Pro.

### Critical appraisal

Two reviewers independently performed a critical appraisal of each included study, through use of the Specialist Unit for Review Evidence (SURE) checklist (2015).^52^ This checklist is adapted and updated from the former Health Evidence Bulletins Wales checklist with reference to the NICE Public Health Methods Manual^58^ and previous versions of the Critical Appraisal Skills Programme checklists. Discrepancies were resolved through discussion between two reviewers. Studies were not excluded or weighted based on the quality assessment.

### Data synthesis

Thematic analysis was the inductive approach used for synthesising the data from each study, an approach used to identify themes and patterns in qualitative research.^59^ This approach has previously been adopted to synthesise qualitative data in systematic reviews.^59–61,62^ Thematic synthesis was completed in three stages: the coding of text “line-by-line”, the generation of “descriptive themes”, and the generation of “analytical themes”.^48^ Initially, the review question was put to one side to enable an analysis that was close to the data of the original studies and prevented reviewers imposing the data on to an existing framework. Participant quotations within the findings/results section of each included study were coded according to meaning and content. A “bank” of codes were derived from the studies, and new codes were formed when necessary. Similarities and differences between the codes were explored, and codes were placed into a hierarchical structure and these represented the descriptive themes. The final stage of analysis involved engaging with the descriptive themes to answer the review question. This was an iterative process, which involved making inferences from the data about the facilitators and barriers to PA following PR, as well as considering implications regarding intervention development. To reduce bias, three reviewers independently coded the extracted data, produced descriptive themes, and reviewed and discussed analytical themes.

### Data availability

No datasets were generated or analysed during the current study.

## Electronic supplementary material

Supplementary file 1

## References

[1] Global Initiative for Chronic Obstructive Lung Disease (GOLD). From the global strategy for the diagnosis, management and prevention of COPD. http://goldcopd.org (2017).

[2] Vorrink SN, Kort HS, Troosters T, Lammers JWJ (2011). Level of daily physical activity in individuals with COPD compared with healthy controls. Respir. Res..

[3] Pitta F (2005). Characteristics of physical activities in daily life in chronic obstructive pulmonary disease. Am. J. Respir. Crit. Care Med..

[4] Arne M (2011). Factors associated with good self-rated health and quality of life in subjects with self-reported COPD. Int. J. Chron. Obstruct. Pulmon. Dis..

[5] Watz H (2014). An official European Respiratory Society statement on physical activity in COPD. Eur. Respir. J..

[6] Decramer M, Janssens W, Miravitlles M (2012). Chronic obstructive pulmonary disease. Lancet.

[7] Vestbo J (2013). Global strategy for the diagnosis, management, and prevention of chronic obstructive pulmonary disease. Am. J. Respir. Crit. Care Med..

[8] Waschki B (2011). Physical activity is the strongest predictor of all-cause mortality in patients with COPD: a prospective cohort study. Chest.

[9] Vaes AW (2014). Changes in physical activity and all-cause mortality in COPD. Eur. Respir. J..

[10] Pitta F (2006). Physical activity and hospitalization for exacerbation of COPD. Chest.

[11] Garcia-Aymerich J, Lange P, Serra I, Schnohr P, Antó JM (2008). Time-dependent confounding in the study of the effects of regular physical activity in chronic obstructive pulmonary disease: an application of the marginal structural model. Ann. Epidemiol..

[12] Garcia-Rio F (2012). Prognostic value of the objective measurement of daily physical activity in patients with COPD. Chest.

[13] Benzo RP (2010). Physical activity, health status and risk of hospitalization in patients with severe chronic obstructive pulmonary disease. Respir. Int. Rev. Thorac. Dis..

[14] Gimeno-Santos E (2014). Determinants and outcomes of physical activity in patients with COPD: a systematic review. Thorax.

[15] Spruit MA (2013). An official American Thoracic Society/European Respiratory Society statement: key concepts and advances in pulmonary rehabilitation. Am. J. Respir. Crit. Care Med..

[16] McCarthy, B. et al. Pulmonary rehabilitation for chronic obstructive pulmonary disease. *Cochrane Database Syst. Rev.* **3**, CD003793 (2015). 10.1002/14651858.CD003793.pub3 PMC10008021 25705944

[17] Mesquita R (2017). Changes in physical activity and sedentary behaviour following pulmonary rehabilitation in patients with COPD. Respir. Med..

[18] Ng LWC, Mackney J, Jenkins S, Hill K (2012). Does exercise training change physical activity in people with COPD? A systematic review and meta-analysis. Chron. Respir. Dis..

[19] Mantoani LC, Rubio N, McKinstry B, MacNee W, Rabinovich RA (2016). Interventions to modify physical activity in patients with COPD: a systematic review. Eur. Respir. J..

[20] Lahham A, McDonald CF, Holland AE (2016). Exercise training alone or with the addition of activity counseling improves physical activity levels in COPD: a systematic review and meta-analysis of randomized controlled trials. Int. J. Chron. Obstruct. Pulmon. Dis..

[21] Beauchamp MK, Evans R, Janaudis-Ferreira T, Goldstein RS, Brooks D (2013). Systematic review of supervised exercise programs after pulmonary rehabilitation in individuals with COPD. Chest.

[22] Rochester CL, Spruit MA (2017). Maintaining the benefits of pulmonary rehabilitation. The holy grail. Am. J. Respir. Crit. Care Med..

[23] Spruit MA, Pitta F, McAuley E, ZuWallack RL, Nici L (2015). Pulmonary rehabilitation and physical activity in patients with chronic obstructive pulmonary disease. Am. J. Respir. Crit. Care Med..

[24] de Sousa Pinto JM (2013). Chronic obstructive pulmonary disease patients’ experience with pulmonary rehabilitation: a systematic review of qualitative research. Chron. Respir. Dis..

[25] Thorpe O, Johnston K, Kumar S (2012). Barriers and enablers to physical activity participation in patients with COPD: a systematic review. J. Cardiopulm. Rehabil. Prev..

[26] Kwasnicka D, Dombrowski SU, White M, Sniehotta F (2016). Theoretical explanations for maintenance of behaviour change: a systematic review of behaviour theories. Health Psychol. Rev..

[27] Hoaas H, Andreassen HK, Lien LA, Hjalmarsen A, Zanaboni P (2016). Adherence and factors affecting satisfaction in long-term telerehabilitation for patients with chronic obstructive pulmonary disease: a mixed methods study. BMC Med. Inform. Decis. Mak..

[28] Hoaas H, Morseth B, Holland AE, Zanaboni P (2016). Are physical activity and benefits maintained after long-term telerehabilitation in COPD?. Int. J. Telerehabilitation.

[29] Beauchamp MK (2012). Feasibility and acceptability of a community based maintenance exercise program for people with COPD. Can. Respir. J..

[30] Desveaux L, Rolfe D, Beauchamp M, Goldstein R, Brooks D (2014). Participant experiences of a community-based maintenance program post-pulmonary rehabilitation. Chron. Respir. Dis..

[31] Langley-Johnson CA (2010). P50 facilitation of continued exercise via patient volunteers with chronic obstructive pulmonary disease (COPD) following a pulmonary rehabilitation programme: a feasibility study. Thorax.

[32] Matheson L (2010). P44 COPD patients derived benefits from attending PR: ‘this has given me my life back’. Thorax.

[33] Camp PG, Appleton J, Reid WD (2000). Quality of life after pulmonary rehabilitation: assessing change using quantitative and qualitative methods. Phys. Ther..

[34] Desveaux L (2017). ‘We are all there for the same purpose’: support for an integrated community exercise program for older adults with HF and COPD. Heart Lung J. Crit. Care.

[35] Halding AG, Heggdal K (2012). Patients’ experiences of health transitions in pulmonary rehabilitation. Nurs. Inq..

[36] Sundfør, I. E. ‘But I May Be Vacuuming…’: A Qualitative Study on Changing Working Habits and Awareness of Energy-Saving Working Methods for People with COPD. Master's Thesis, http://hdl.handle.net/10642/556 (Oslo University College, 2010).

[37] Stewart KFJ (2014). Maintenance of a physically active lifestyle after pulmonary rehabilitation in patients with COPD: a qualitative study toward motivational factors. J. Am. Med. Dir. Assoc..

[38] Hogg L, Grant A, Garrod R, Fiddler H (2012). People with COPD perceive ongoing, structured and socially supportive exercise opportunities to be important for maintaining an active lifestyle following pulmonary rehabilitation: a qualitative study. J. Physiother..

[39] Lewis R, Cramp F (2010). Facilitators and barriers to exercise maintenance in chronic obstructive pulmonary disease: patient views. Physiother. Pract. Res..

[40] Rodgers S, Dyas J, Molyneux AWP, Ward MJ, Revill SM (2007). Evaluation of the information needs of patients with chronic obstructive pulmonary disease following pulmonary rehabilitation: a focus group study. Chron. Respir. Dis..

[41] Williams V, Bruton A, Ellis-Hill C, McPherson K (2010). The effect of pulmonary rehabilitation on perceptions of breathlessness and activity in COPD patients: a qualitative study. Prim. Care Respir. J..

[42] Norweg A, Bose P, Snow G, Berkowitz ME (2008). A pilot study of a pulmonary rehabilitation programme evaluated by four adults with chronic obstructive pulmonary disease. Occup. Ther. Int..

[43] Rabinowitz, M. C. Stories of Exercise Noncompliane among Patients with Chronic Obstructive Pulmonary Disease after Completion of Pulmonary Rehabilitation. PhD Thesis, http://link.library.missouri.edu/portal/Stories-of-exercise-noncompliance-among-patients/b2ze39d84hw/ (Adelphi University, 1998).

[44] Zakrisson AB, Theander K, Anderzén-Carlsson A (2014). How life turned out one year after attending a multidisciplinary pulmonary rehabilitation programme in primary health care. Prim. Health Care Res. Dev..

[45] Granger CL (2017). Understanding factors influencing physical activity and exercise in lung cancer: a systematic review. Support. Care Cancer.

[46] Michie, S., Atkins, L. & West, R. *The behaviour change wheel: a guide to designing interventions.* (London, Silverback publishing, 2014).

[47] Britten N (2002). Using meta ethnography to synthesise qualitative research: a worked example. J. Health Serv. Res. Policy.

[48] Thomas, J. & Harden, A. Methods for the thematic synthesis of qualitative research in systematic reviews. *BMC Med. Res. Methodol.* **8**, 45 (2008). 10.1186/1471-2288-8-45 PMC2478656 18616818

[49] Campbell R (2003). Evaluating meta-ethnography: a synthesis of qualitative research on lay experiences of diabetes and diabetes care. Soc. Sci. Med..

[50] Sandelowski, M. & Barroso, J. *Handbook for synthesizing qualitative research*. (New York, Springer, 2007).

[51] Tong, A., Flemming, K., McInnes, E., Oliver, S. & Craig, J. Enhancing transparency in reporting the synthesis of qualitative research: ENTREQ. *BMC Med. Res. Methodol.* **12**, 181 (2012). 10.1186/1471-2288-12-181 PMC3552766 23185978

[52] Specialist Unit for Review Evidence (SURE). Questions to assist with the critical appraisal of qualitative studies. http://www.cardiff.ac.uk/specialist-unit-for-review-evidence/resources/critical-appraisalchecklists (2015).

[53] Dixon-Woods M (2007). Appraising qualitative research for inclusion in systematic reviews: a quantitative and qualitative comparison of three methods. J. Health Serv. Res. Policy.

[54] Effing, T. et al. Self-management education for patients with chronic obstructive pulmonary disease. *Cochrane Database of Syst. Rev.* **4**, CD002990 (2007). 10.1002/14651858.CD002990.pub2 17943778

[55] Moher D (2015). Preferred reporting items for systematic review and meta-analysis protocols (PRISMA-P) 2015 statement. Syst. Rev..

[56] Caspersen CJ, Powell KE, Christenson GM (1985). Physical activity, exercise, and physical fitness: definitions and distinctions for health-related research. Public Health Rep..

[57] National Collaborating Centre for Mental Health (UK). Dementia: A NICE-SCIE Guideline on Supporting People With Dementia and Their Carers in Health and Social Care. (British Psychological Society, 2007). 21834193

[58] National Institute for Health and Care Excellence: The guidelines manual. Process and methods: https://www.nice.org.uk/process/pmg6/chapter/introduction (2012). 27905714

[59] Braun V, Clarke V (2006). Using thematic analysis in psychology. Qual. Res. Psychol..

[60] Franco, M. R. et al. Older people’s perspectives on participation in physical activity: a systematic review and thematic synthesis of qualitative literature. *Br. J. Sports Med.* **49**, 1268–1276 (2015). 10.1136/bjsports-2014-094015 25586911

[61] Morton RL, Tong A, Howard K, Snelling P, Webster AC (2010). The views of patients and carers in treatment decision making for chronic kidney disease: systematic review and thematic synthesis of qualitative studies. Br. Med. J..

[62] Joseph-Williams N, Elwyn G, Edwards A (2014). Knowledge is not power for patients: a systematic review and thematic synthesis of patient-reported barriers and facilitators to shared decision making—ScienceDirect. Patient Educ. Couns..

