# Supplementary material for: Facilitators and barriers to physical activity following pulmonary rehabilitation in COPD: a systematic review of qualitative studies
Source: NPJ Prim Care Respir Med. 2018 Jun 4;28:19. doi: 10.1038/s41533-018-0085-7 (PMC5986863; doi:10.1038/s41533-018-0085-7)
Supplement: Supplementary file 1 [file 41533_2018_85_MOESM1_ESM.pdf]

Supplementary file 1

Table: Excluded studies with justifications for exclusion.

	Study reference	Exclusion based on: Study design, Population, Outcome
1	Apps et al., 2013 [1]	Population: History of PR participation not reported (Conference abstract)
2	Arnold, Bruton, & Ellis-Hill, 2006 [2]	Population: Not all individuals had completed PR<br>Outcome: Focus on experiences during PR only
3	Beauchamp et al., 2012 [3]	Data already included in review
4	Burge et al., 2013 [4]	Population: COPD patients could not be distinguished from other participants<br>Outcome: No discussion of PA
5	Caress, Chalmers, & Luker, 2010 [5]	Population: Not all individuals had completed PR
6	De Sousa Pinto et al., 2013 [6]	Study design: Systematic review
7	Desveaux, Goldstein, Mathur, & Brooks, 2016 [7]	Outcome: Quantitative data only
8	Fabienne Dobbels et al., 2014 [8]	Population: History of PR not reported
9	Fabienne Dobbels et al., 2011[9]	Population: History of PR not reported<br>(Conference abstract)
10	Halding, Wahl, & Heggdal, 2010 [10]	Outcomes: No discussion of PA
11	Hamir et al., 2012 [11]	Outcome: No discussion of the barriers and facilitators to PA

	Study reference	Exclusion based on: Study design, Population, Outcome
12	Hardy & Coe, 2011 [12]	Outcome: No discussion of the barriers and facilitators to PA (Conference abstract)
13	Hartman, ten Hacken, Boezen, & de Greef, 2013 [13]	Outcomes: No discussion of PA
14	Lahham et al., 2015 [14]	Outcome: Focus on experiences during PR only
15	Langley-Johnson et al., 2010 [15]	Conference abstract without access to primary data<br>Mentioned in the discussion
16	Larson, Fernandez, & Vos, 2015 [16]	Population: History of participation in PR not reported
17	Leidy & Haase, 1996 [17]	Population: History of participation in PR not reported
18	Matheson et al., 2010 [18]	Conference abstract without access to primary data<br>Mentioned in the discussion
19	Meis et al., 2014 [19]	Population: Not completed PR
20	Nonoyama, Holmes, King, & Brooks, 2010 [20]	Population: No distinction between participants with COPD and participants with Asthma<br>Outcome: Quantitative data only
21	O'Connor et al., 2009 [21]	Population: No participation in PR
22	O'Shea, Taylor, & Paratz, 2007 [22]	Population: No participation in PR

	Study reference	Exclusion based on:<br>Study design, Population, Outcome
23	Pillard, 2014 [23]	Outcome: Quantitative data only
24	Poureslami et al., 2017 [24]	Population: Not all individuals had completed PR
25	Small et al., 2012 [25]	Population: No history of participation in PR
26	Thomas, Williams, & Stern, 2015 [26]	Population: Not exclusively COPD patients<br>Outcome: No discussion of PA<br>(Conference abstract)
27	Thorpe, Kumar, & Johnston, 2014 [27]	Population: Not participated in PR
28	Valenson et al., 2016 [28]	Population: Not participated in PR
29	Verwey et al., 2014 [29]	Population: No history of participation in PR
30	Walters et al., 2012 [30]	Population: Not participated in PR
31	Wang et al., 2013 [31]	Population: Not all individuals had completed PR
32	Williams, Hardinge, Ryan, & Farmer, 2014 [32]	Population: Not all individuals had completed PR
33	Wong et al., 2014 [33]	Study design: Quantitative data only
34	Wortz et al., 2012 [34]	Population: No history of PR completion
35	Yang & Chen, 2005 [35]	Outcome: Quantitative data only
36	Yorke et al., 2012 [36]	Conference poster: Restricted access to primary data

	Study reference	Exclusion based on: Study design, Population, Outcome
37	Young et al., 2014 [37]	Population: History of PR not reported
38	Zanaboni et al., 2016 [38]	Population: Not participated in PR

Reference list

1. Apps, L. D. *et al.* The development and pilot testing of the Self-management Programme of Activity, Coping and Education for Chronic Obstructive Pulmonary Disease (SPACE for COPD). *Int. J. Chron. Obstruct. Pulmon. Dis.* **8,** 317–327 (2013).
2. Arnold, E., Bruton, A. & Ellis-Hill, C. Adherence to pulmonary rehabilitation: A qualitative study. *Respir. Med.* **100,** 1716–1723 (2006).
3. Beauchamp, M., K. *et al.* Feasibility and acceptability of a community based maintenance exercise program for people with COPD. *Can. Respir. J.* **19,** e42 (2012).
4. Burge, A. T. *et al.* Advance care planning education in pulmonary rehabilitation: A qualitative study exploring participant perspectives. *Palliat. Med.* **27,** 508–515 (2013).
5. Caress, A., Luker, K. & Chalmers, K. Promoting the health of people with chronic obstructive pulmonary disease: patients' and carers' views. *J. Clin. Nurs.* **19,** 564–573 (2010).
6. de Sousa Pinto, J. M. *et al.* Chronic obstructive pulmonary disease patients' experience with pulmonary rehabilitation: a systematic review of qualitative research. *Chron. Respir. Dis.* **10,** 141–157 (2013).
7. Desveaux, L. *et al.* Effects of a Community-Based, Post-Rehabilitation Exercise Program in COPD: Protocol for a Randomized Controlled Trial With Embedded Process Evaluation. *JMIR Res. Protoc.* **5,** (2016).

8. Dobbels, F. *et al.* The PROactive innovative conceptual framework on physical activity. *Eur. Respir. J.* **44,** 1223–1233 (2014).
9. Dobbels, F. *et al.* A new model to understand the concept physical activity for patients with COPD. *Eur. Respir. J.* **38,** p291 (2011).
10. Halding, A.-G., Wahl, A. & Heggdal, K. 'Belonging'. 'Patients'' experiences of social relationships during pulmonary rehabilitation'. *Disabil. Rehabil.* **32,** 1272–1280 (2010).
11. Hamir, R. *et al.* Patient Evaluation Of A Peer Educator Vs. Respiratory Therapist Support Program Aimed At Maintaining Physical Activity Following Pulmonary Rehabilitation. *Am J Respir* Crit Care *Med*. **185**, A4883–A4883 (2012).
12. Hardy, A. & Coe, A. Participants perspectives of pulmonary rehabilitation: The role of peer support. *Eur. Respir. J.* **38,** 3657 (2011).
13. Hartman, J. E., ten Hacken, N. H. T., Boezen, H. M. & de Greef, M. H. G. Self-efficacy for physical activity and insight into its benefits are modifiable factors associated with physical activity in people with COPD: A mixed-methods study. *J. Physiother.* **59,** 117–124 (2013).
14. Lahham, A. *et al.* P100 Home-based pulmonary rehabilitation in chronic obstructive pulmonary disease: the patient experience. *Nurs. Sig- Poster Present.* **20,** 62–158 (2015).
15. Langley-Johnson, C. A. *et al.* P50 Facilitation of continued exercise via patient volunteers with chronic obstructive pulmonary disease (COPD) following a pulmonary rehabilitation programme: a feasibility study. *Thorax* **65,** A98 (2010).
16. Larson, J. L., Fernandez, D. & Vos, C. M. Chronic obstructive pulmonary disease: Perceptions of physical activity. *Am J Respir Crit Care Med.* **191**, A2000 (2015).
17. Leidy, N. K. & Haase, J. E. Functional Performance in People with Chronic Obstructive Pulmonary Disease: A Qualitative Analysis: *Adv. Nurs. Sci.* **18,** 77–89 (1996).
18. Matheson, L. *et al.* P44 COPD Patients derived benefits from attending PR: 'This has given me my life back'. *Thorax* **65,** A95–A95 (2010).

19. Meis, J. J. M. *et al.* A qualitative assessment of COPD patients' experiences of pulmonary rehabilitation and guidance by healthcare professionals. *Respir. Med.* **108,** 500–510 (2014).
20. Nonoyama, M, L, Holmes, R, King, J & Brooks, D. Lung Association's Ottawa COPD program: A successful maintenance pulmonary rehabilitation program. *Can. J. Respir. Ther.* **46,** 18–23 (2010).
21. O'Connor, J. *et al.* COPD patients' beliefs and expectations of pulmonary rehabilitation. *Thorax.* **64,** A97-98 (2009).
22. O'Shea, S. D., Taylor, N. F. & Paratz, J. D. . . . But watch out for the weather: factors affecting adherence to progressive resistance exercise for persons with COPD. *J. Cardiopulm. Rehabil. Prev.* **27,** 166–174 (2007).
23. Pillard, F. Faciliter et stimuler l'activité physique des patients atteints de BPCO : quels moyens, quels résultats? *Rev. Mal. Respir. Actual.* **6,** 235–237 (2014).
24. Poureslami, I. *et al.* Using Exploratory Focus Groups to Inform the Development of a Peer-Supported Pulmonary Rehabilitation Program: Directions for further research. *J. Cardiopulm. Rehabil. Prev.* **37,** 57–64 (2017).
25. Small, N. *et al.* 'You get old, you get breathless, and you die': chronic obstructive pulmonary disease in Barnsley, UK. *Health Place* **18,** 1396–1403 (2012).
26. Thomas, R., Williams, H. & Stern, M. P137 'I really live for coming here'. The effect of a long-term singing group on control of breathlessness, social empowerment and psychological wellbeing of patients with respiratory disease: a qualitative study. *Thorax.* **70,** A145.1-A145 (2015).
27. Thorpe, O., Kumar, S. & Johnston, K. Barriers to and enablers of physical activity in patients with COPD following a hospital admission: a qualitative study. *Int. J. Chron. Obstruct. Pulmon. Dis.* **9,** 115–128 (2014).
28. Valenson, W. *et al.* Perceived Barriers to Physical Activity in Patients at High Risk for COPD Exacerbations. *Chest* **150,** 892A (2016).

29. Verwey, R. *et al.* A pilot study of a tool to stimulate physical activity in patients with COPD or type 2 diabetes in primary care. *J. Telemed. Telecare* **20,** 29–34 (2014).

30. Walters, J. A. E. *et al.* Supporting health behaviour change in chronic obstructive pulmonary disease with telephone health-mentoring: insights from a qualitative study. *BMC Fam. Pract.* **13,** (2012).

31. Wang, H.-C. *et al.* An exploration of beliefs regarding exercise among Taiwanese patients with chronic obstructive pulmonary disease. *Heart Lung J. Acute Crit. Care* **42,** 133–138 (2013).

32. Williams, V., Hardinge, M., Ryan, S. & Farmer, A. Patients' experience of identifying and managing exacerbations in COPD: a qualitative study. *Npj Prim. Care Respir. Med.* **24,** (2014).

33. Wong, E. Y. *et al.* Peer educator vs. respiratory therapist support: which form of support better maintains health and functional outcomes following pulmonary rehabilitation? *Patient Educ. Couns.* **95,** 118–125 (2014).

34. Wortz, K. *et al.* A qualitative study of patients' goals and expectations for self-management of COPD. *Prim. Care Respir. J.* **21,** 384–391 (2012).

35. Yang, P.-S. & Chen, C.-H. Exercise Stage and Processes of Change in Patients With Chronic Obstructive Pulmonary Disease: *J. Nurs. Res.* **13,** 97–105 (2005).

36. Yorke, J *et al.* Rehabilitative electronic assistance for COPD in the home (REACH): A feasibility study. *Am. J. Respir. Crit. Care Med.* **185,** A4872 (2012).

37. Young, J. *et al.* M148 Investigating The Feasibility Of An Online Health Resource With Nurse Coaching To Support Self-management In Copd. *Thorax* **69,** A217–A218 (2014).

38. Zanaboni, P. *et al.* Long-term integrated telerehabilitation of COPD Patients: a multicentre randomised controlled trial (iTrain). *BMC Pulm. Med.* **16,** 126 (2016).

Supplementary file 2

Table: Example of a search strategy used in MEDLINE

Medline	Search term	Field
1	(MH"Lung disease, Obstructive")	MH
2	(MH "Pulmonary Disease, Chronic Obstructive+")	MH (Explode)
3	"COPD"	TX
4	"COAD"	TX
5	"COBD"	TX
6	"AECB"	TX
7	"Emphysem*"	TX
8	"Chronic N3 bronchit*"	TX
9	"Obstruct* N3 airflow*" OR "airway*" OR "bronch*" OR "lung*" OR "pulmonary" OR "respirator*"	TI, AB
10	1 OR 2 OR 3 OR 4 OR 5 OR 6 OR 7 OR 8 OR 9	
11	Exercise	(MH "Exercise+")
12	Exercise movement techniques	(MH "Exercise Movement Techniques+")
13	Physical and rehabilitation medicine	(MH "Physical and Rehabilitation Medicine+")
14	(MH "Physical fitness")	MH
15	Exercise therapy	(MH "Exercise Therapy+")
16	Activities of daily living	(MH "Activities of Daily Living+")
17	Self-care	(MH "Self Care+")
18	Physical endurance	(MH "Physical Endurance+")
19	Health behavior	(MH "Health Behavior+")
20	Health N3 behav*	TX
21	Physical* N3 activ*	TX
22	Physical* N3 rehabilitat*	TX

23	Physical* N3 mobil*	TX
24	Physical* N3 fit*	TX
25	Endurance	TX
26	maintain* N3 exercis*	TI/AB
27	maintain N3 activ*	TI/AB
28	“Program*” or “training”	TI/AB
29	11 OR 12 OR 13 OR 14 OR 15 OR 16 OR 17 OR 18 OR 19 OR 20 OR 21 OR 22 OR 23 OR 24 OR 25 OR 26 OR 27 OR 28	
30	Facilitat*	TI/AB
31	Enabl*	TI/AB
32	Barrier*	TI/AB
33	Hinder*	TI/AB
34	Overcom*	TI/AB
35	Promot*	TI/AB
36	Limit*	TI
37	Support*	TI
38	30 OR 31 OR 32 OR 33 OR 34 OR 35 OR 36 OR 37	
39	Qualitative research	(MH "Qualitative Research+")
40	Interview	(MH "Interview")
41	Focus groups	(MH "Focus Groups")
42	Qualitativ*	TI, AB
43	Interview*	TI,AB
44	Mix* N3 method*	TX
45	Process* N3 eval*	TX
46	Program* N3 eval*	TX
47	Method* N3 triangulat*	TX
48	Focus group*	TX
49	Ethnograph*	TX
50	Phenomenol*	TX
51	Ground* N3 theor*	TX
52	Discourse analys*	TX
53	Purposive	TX
54	Narrative*	TX

55	Content* N3 analys*	TX
56	thematic	TX
57	Verbatim	TX
58	Theme*	TX
59	Belief*	TI
60	39 OR 40 OR 41 OR 42 OR 43 OR 44 OR 45 OR 46 OR 47 OR 48 OR 49 OR 50 OR 51 OR 52 OR 53 OR 54 OR 55 OR 56 OR 57 OR 58 OR 59	
61	10 AND 29 AND 38 AND 60	
